# PDTD: a web-accessible protein database for drug target identification

**DOI:** 10.1186/1471-2105-9-104

**Published:** 2008-02-19

**Authors:** Zhenting Gao, Honglin Li, Hailei Zhang, Xiaofeng Liu, Ling Kang, Xiaomin Luo, Weiliang Zhu, Kaixian Chen, Xicheng Wang, Hualiang Jiang

**Affiliations:** 1 Drug Discovery and Design Center, State Key Laboratory of Drug Research, Shanghai Institute of Materia Medica, Chinese Academy of Sciences, Shanghai 201203, China; 2 Department of Engineering Mechanics, State Key Laboratory of Structural Analysis for Industrial Equipment, Dalian University of Technology, Dalian 116023, China; 3 School of Pharmacy, East China University of Science and Technology, Shanghai 200237, China

## Abstract

**Background:**

Target identification is important for modern drug discovery. With the advances in the development of molecular docking, potential binding proteins may be discovered by docking a small molecule to a repository of proteins with three-dimensional (3D) structures. To complete this task, a reverse docking program and a drug target database with 3D structures are necessary. To this end, we have developed a web server tool, TarFisDock (*Tar*get *Fis*hing *Dock*ing) , which has been used widely by others. Recently, we have constructed a protein target database, *P*otential *D*rug *T*arget *D*atabase (PDTD), and have integrated PDTD with TarFisDock. This combination aims to assist target identification and validation.

**Description:**

PDTD is a web-accessible protein database for *in silico *target identification. It currently contains >1100 protein entries with 3D structures presented in the Protein Data Bank. The data are extracted from the literatures and several online databases such as TTD, DrugBank and Thomson Pharma. The database covers diverse information of >830 known or potential drug targets, including protein and active sites structures in both PDB and mol2 formats, related diseases, biological functions as well as associated regulating (signaling) pathways. Each target is categorized by both nosology and biochemical function. PDTD supports keyword search function, such as PDB ID, target name, and disease name. Data set generated by PDTD can be viewed with the plug-in of molecular visualization tools and also can be downloaded freely. Remarkably, PDTD is specially designed for target identification. In conjunction with TarFisDock, PDTD can be used to identify binding proteins for small molecules. The results can be downloaded in the form of mol2 file with the binding pose of the probe compound and a list of potential binding targets according to their ranking scores.

**Conclusion:**

PDTD serves as a comprehensive and unique repository of drug targets. Integrated with TarFisDock, PDTD is a useful resource to identify binding proteins for active compounds or existing drugs. Its potential applications include *in silico *drug target identification, virtual screening, and the discovery of the secondary effects of an old drug (i.e. new pharmacological usage) or an existing target (i.e. new pharmacological or toxic relevance), thus it may be a valuable platform for the pharmaceutical researchers. PDTD is available online at .

## Background

Until 2000, only ~500 drug targets had been reported [1], among which only 120 drug targets are actually marketed [2]. The completion of human genome and numerous pathogen genomes suggests that there are 30,000 to 40,000 genes and at least the same number of proteins, and many of these proteins are potential targets for drug discovery. It has been estimated that there are more than 2,000 potential drug targets with at least one drug candidate in clinical trial [2,3]. This is a reservoir for drug discovery and target identification. However, how to extensively utilize this source is a challenge. Expressing all these proteins and screening compounds against the corresponding models constructed based on the proteins is extremely unpractical, because it is intolerably expensive and time-consuming. Recent promising advancement in docking-based virtual screening has demonstrated the efficiency of this approach in discovering lead (active) compounds [4,5]. On the other hand, reverse (or inverse) docking approaches have become promising computational tools to find the probable target proteins for active compounds, natural products or old drugs [6-10]. Both these two researches need the information of target proteins, in particular the information of structures and active sites. However, such information of most drug targets is dispersedly deposited in the literatures or other databases like Protein Data Bank (PDB). Therefore, it is in dire need of a database containing comprehensive information of the potential target proteins.

Recently, some notable efforts have been made to partially satisfy this requirement. The Therapeutic Target Database (TTD) is one such example [11], which provides information about the known therapeutic targets, disease conditions and the corresponding drugs. DrugBank is a bioinformatics/cheminformatics resource that combines detailed drug data with comprehensive drug target information [12]. A number of ligand-protein interaction databases have also emerged including LigBase [13], PDBsite [14], SitesBase [15], MSDsite [16], PDB-Ligand [17] and AffinDB [18]. Unfortunately, these databases were not specifically designed for discovering new leads by using virtual screening approaches and new targets by using reverse docking. They also cannot be used to figure out specific pharmaceutical information related to the secondary effects of an old drug (i.e. new pharmacological usage) or an existing target (i.e. new pharmacological or toxic relevance). Ideally, a target database may provide not only abundant information about the potential target proteins such as 3D structures, binding (active) sites, biological (pharmacological) functions, related diseases, but also appropriate computational tools to mine the information about targets. Herein, we present a web-accessible protein database, PDTD (*P*otential *D*rug *T*arget *D*atabase). Integrated with our reverse docking server, TarFisDock [8], PDTD is a valuable platform for target identification.

## Construction and content

**Figure 1:**
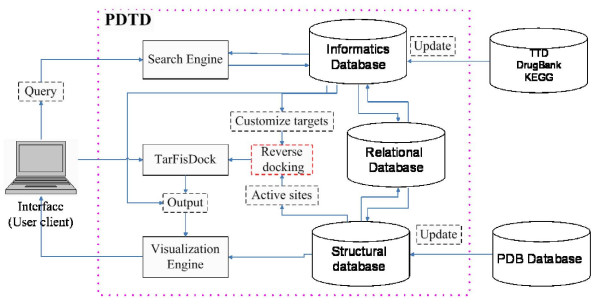
**PDTD system architecture**. The system is implemented in MySQL and PHP script, user can freely access the database at .

Fundamentally, PDTD has dual functions of querying drug target information and identifying the potential binding proteins of an active compound or an existing drug by using reverse docking approach. Accordingly, PDTD contains two sub-databases types, one is the structural sub-database and another is the informatics sub-database. All data are associated with a relational database implemented using MySQL and can be queried through web interface. Through three computational engines, search engine, visualization engine and TarFisDock, users can implement interactive query and computation with the PDTD (Figure 1). The structural sub-database stores each protein in both PDB format and mol2 format with Amber charges; sequence and active site information were also included in the structural sub-database. The informatics database stores the data of target categories, related disease information, biological functions and associated regulating (signaling) pathways. PDTD currently contains >1100 entries covering the information of >800 known drug targets.

The target proteins in PDTD were selected from scientific literatures [1,19-21] and several online databases such as TTD [11], DrugBank [12] and Thomson Pharma [22]. Since PDTD is designed to search the probable binding proteins for new active compounds or existing drugs by using reverse docking, it only contains the proteins with known 3D structures determined experimentally by the X-ray crystallographic or NMR methods. The coordinates of proteins were isolated from the PDB. Since not all PDB structures are of equal quality, a protein structure is selected according to the following criteria when it has several redundant records in PDB: (i) select the structure without mutation and missing residues around the active site; (ii) select the structure with high resolution; (iii) select the structure complexed with ligand. For each selected protein in PDTD, amino acid residues within 6.5 Å around the bound ligand were used to define the binding (active) site. A PDB entry could contain data on a number of binding sites. If so, a separate entry was generated in the PDTD to accommodate each of the sites. HETATM records in PDB files were used to define the ligands. PDB and mol2 files of each protein were also stored in the structural sub-database. All kinds of structures for a drug target can be visualized using the "Jmol" JAVA applet [23].

**Figure 2:**
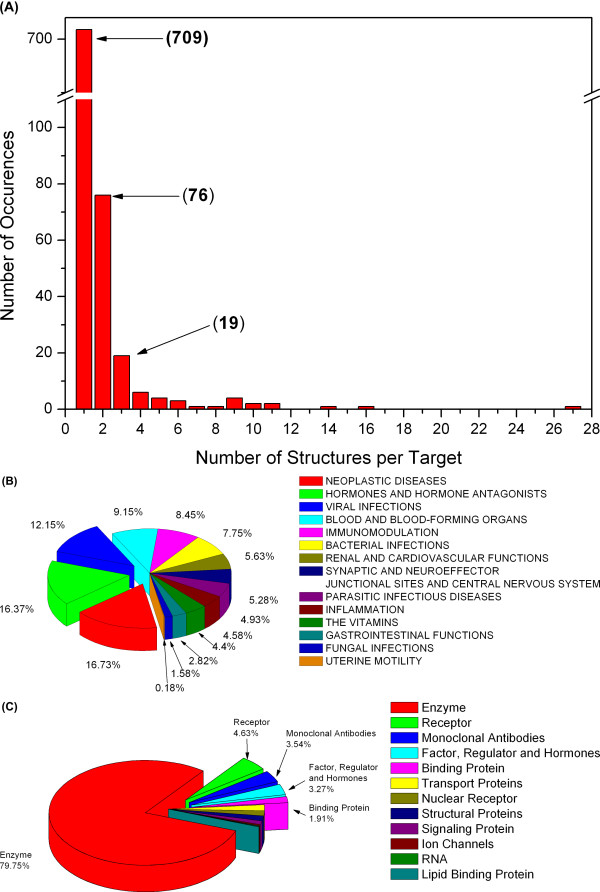
**Functional and biochemical classifications of PDTD protein entries**. (A) Database redundancy. Each bar represents the number of targets that have the same amounts of copies in the PDTD. Break is applied to the *y *axes. (B) Distribution of drug targets according to their therapeutic areas. (C) Distribution of drug targets according to their biochemical criteria, which include enzymes, receptors, ion channels, transporters, nuclear receptors, binding protein, structural proteins, signaling protein, and factors, regulators and hormones.

Most of drug targets in PDTD have been collected with single structure (709 cases). Since our reverse docking program, TarFisDock, has not considered the flexibility of proteins, PDTD contains some redundancy for the flexible proteins. For example, HIV-1 protease has 27 entries and dihydrofolate reductase (DHFR) has 14 entries in the database. The redundancy of each target is shown in Figure 2a.

According to therapeutic areas, the drug targets may be categorized into 14 types (Figure 2b). It is convenient for users to custom a special list when they predict potential binding targets for small molecules using TarFisDock. Among targets having explicit therapeutic functions, the targets related neoplastic disease are most populated, following are hormones and hormones antagonists related targets. Targets related to viral infections are also major contributors in PDTD. The distribution of biochemical classification is shown in Figure 2c, indicating that PDTD mainly consists of enzymes, receptors, ion channels, transporters, nuclear receptors, binding protein, structural proteins, signaling proteins, factors, regulators and hormones. The targets which can not be assigned into any of these biochemical classes are grouped into an "unknown" class. The selected drug targets are enriched in enzymes (80.2%). G-protein coupled receptors (GPCR) and other receptors which account for most drug targets seldom have crystal structures, resulting that the ratio of receptor targets in PDTD is only 4.2%.

## Utility and Discussion

### Web interface: query, download and exploration

**Figure 3:**
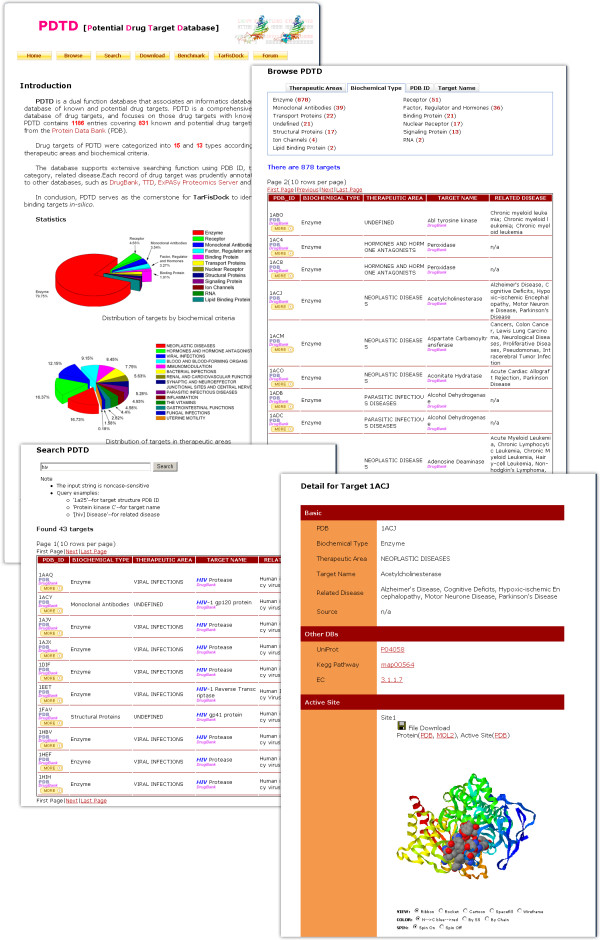
**Screen shots of the PDTD**. A screen shot of the PDTD showing several possible view of information describing the drug target. Not all fields are shown.

PDTD is supported with a friendly designed web interface so that users can easily query the target information, and retrieve, visualize or download the distributions of the drug target files as they desire (Figure 3). PDTD has been designed to provide fast and easy access to target information. The popular MySQL backend was chosen as database server. Using the scripting language PHP, special care was taken to generate a clearly structured layout which enables fast and easy navigation.

All the data can be accessed and retrieved directly via the web browser, PDTD consists of a classification table and a keyword search box. The user can search a drug target manually from the classification table, or automatically by using the keyword search function, such as PDB ID, target name, or disease. Every target has its own result page containing comprehensive information including PDB ID, target name, target category, related disease, its structure, and active site. The PDTD was carefully annotated according to information found in the PDB, UniProt [24], KEGG [25] and Enzyme Structures Database [26]. PDTD also provides hyperlinks to other databases like TTD and DrugBank, which allow easy navigation for more information about target structure, source and function (See *Links to other databases*). The related structures for each drug target can be downloaded freely from the detailed page by clicking on "MORE" button. Furthermore, user can download the classified target structures and all target files from the "Download" page.

Also, users can customize the list of drug targets in which they want to perform reverse docking process to predict the potential binding targets for any small molecule, which to our knowledge is not provided by other public websites. Consequently, TarFisDock will output the list of results with binding poses of molecules against each targets, along with corresponding information, disease, annotation and links to other databases, which are also presented (See the *Applications *below).

### Applications

In bringing together the reverse docking server TarFisDock [8], PDTD has been widely used to identify binding proteins for small molecule. The binding proteins for several molecules have been verified through bioassay and crystal structure determination for target-ligand complexes [9]. In general, one drug molecule may interact with several targets including targets associated with side effect (toxicity). TarFisDock provides multiple options for selecting protein targets. These clues are useful for further experimental test in discovering new pharmacological efficacy or toxicity for an existing drug. In general, combining with PDTD, TarFisDock web sever is a convenient tool for "fishing" the target proteins of small molecules, the user just inputs the structure of querying compound and customizes a target list from PDTD (a list of all the targets is recommended). The results can be downloaded in the form of mol2 file with the binding pose of each compound and a list of potential binding targets according to their ranking scores.

In addition, benchmark searches for the old version of PDTD (698 entries) were performed using TarFisDock taking vitamin E, an anti-oxidant, and 4H-tamoxifen, an anti-cancer agent as probes [8]. In this study, similar benchmark searches for the current version of PDTD (1186 entries) have been carried out. For vitamin E, eight (12 entries) of the twelve experimentally verified targets fall into the top 10% candidates picked up from the PDTD by the TarFisDock program. For 4H-tamoxifen, five (14 entries) of the eleven experimentally confirmed targets appear amongst the top 10% of the TarFisDock predicted candidates. In addition, the PDTD was searched by the TarFisDock using *N*-*trans*-caffeoyltyramine (compound 1), an active natural product discovered by anti-*H. pylori *screening in our lab, as a probe in the previous research [9]. Homology search revealed that, among the fifteen candidates discovered by reverse docking, diaminopimelate decarboxylase (DC) and peptide deformylase (PDF) are possible binding proteins of compound 1. Enzymatic assay demonstrated compound 1 and its derivative compound 2 are the potent inhibitors against the *H. pylori *PDF (*Hp*PDF) with IC_50 _values of 10.8 and 1.25 μM, respectively. X-ray crystal structures of *Hp*PDF and the complexes of *Hp*PDF with 1 and 2 were determined, indicating that these two inhibitors bind well with the *Hp*PDF binding pocket [9]. To exemplify the applications of PDTD combining with TarFisDock, the brief results of these three benchmark examples have been uploaded to the PDTD homepage under the "Benchmark" option.

### Links to other databases

General links are given to related drug and target information with other databases [11,12]. Each data in PDTD is linked to the Protein Data Bank, DrugBank, there are also hypertext links to UniProt [24], Kegg [25] and Enzyme Structures Database [26] for further structural and functional information.

## Conclusion

In summary, PDTD is a comprehensive, web-accessible database of drug targets, which focuses on those drug targets with known 3D-structures. By far, PDTD has collected >1100 entries covering >800 known and potential drug targets from the Protein Data Bank. PDB structure, mol2 file and active site information of each drug target were extracted from the crystal structure, and all the information can be viewed with molecular visualization tools and can be downloaded freely by users. Drug targets of PDTD were categorized by two criteria: therapeutic areas and biochemical criteria. Each target was carefully annotated by browsing several databases, such as DrugBank, TTD, and UniProt. All these information were stored in informatics sub-database, which was associated to structural sub-database with a relational database. Users can also use our reverse docking program to search PDTD for finding the possible binding protein(s) of a small molecule.

One drug molecule may interact with several targets including targets associated with side effect (toxicity). By searching PDTD, TarFisDock may provide multiple options of the binding proteins for a small molecule. These clues are useful for further experimental test in discovering new targets and new pharmacological efficacy or toxicity for an existing drug. Thus, combining with TarFisDock, PDTD is a good web-accessible protein database for identifying drug targets and for discovering new usages of old drugs [27,28]. The user just inputs the structure of querying compound and customizes a target list from PDTD (a list of all the targets is recommended), TarFisDock may provide possible binding proteins of the compound. The results can be downloaded in the form of mol2 file with the binding pose of each compound and a list of potential binding targets according to their ranking scores.

PDTD will be updated continuously. We intend to classify the drug targets according more completely to their biological functions, which will be achieved by integrating and/or linking PDTD with other bioinformatics databases. For example, links can be directed to the databases of SOURCE [29] and Gene Ontology [30] for more descriptions of functional annotations, ontologies, and gene expression data.

## Availability and requirements

PDTD is freely available for academic user at . To download the files of PDTD, users must complete a simple registration process and agree not to republish the data without explicit permission. Users are invited to contact us through the 'Contact' link and to participate in the user forum at .

## Authors' contributions

ZG developed the web interface, designed the relational database scheme, and integrated the database-PDTD with the reverse docking program-TarFisDock. HL developed the reverse docking program, participated in the design of web interface, and contributed to writing the manuscript. HZ, XL, and LK were major data contributors of the current system. XL, WZ and KC provided comments and suggestions about the features of the database. XW and HJ conceived the idea of the database, provided direction for its development and revised the subsequent drafts of this manuscript. All authors read and approved the final manuscript.
